# Supplementary material for: High Flow Oxygen Therapy at Two Initial Flow Settings versus Conventional Oxygen Therapy in Cardiac Surgery Patients with Postextubation Hypoxemia: A Single-Center, Unblinded, Randomized, Controlled Trial
Source: J Clin Med. 2021 May 12;10(10):2079. doi: 10.3390/jcm10102079 (PMC8151420; doi:10.3390/jcm10102079)
Supplement: Supplementary file 1 [file jcm-10-02079-s001.zip › jcm-1204892-supplementary.pdf]

Supplementary materials

1. Supplemental Methods

For high flow nasal canula (HFNC) respiratory support, the AIRVO™ 2 (Fisher & Paykel Healthcare, Auckland New Zealand) with built in flow generator was used.[1] Briefly, standard HFNC device features include an air-oxygen blender enabling delivery of FiO_2s of 0.2 to 1.0 at flow rates of ≤60 L/min, a heated humidifier, a nasal cannula, and an oxygen analyzer regularly calibrated according to manufacturer instructions [1]. HFNC was administered through medium or large size nasal prongs depending on the size of patient's nares [1]. All patients of the Intervention groups were advised to breathe with their mouths closed.

Statistical Analysis – additional details

Type of operation and duration of ischemia were not included in the Cox model described in the main paper, because of moderate-to-very strong linear relationships[2] (r = 0.59 and r = 0.86, respectively) with cardiopulmonary bypass time. Duration of postoperative endotracheal intubation was not included in the model, due to a very strong linear relationship (r = 0.80) with the duration of postoperative sedation. Colinearity was assessed by determination of the condition index, and the variance inflation index. The proportional hazards assumption was tested by visual inspection of the "log minus log plot".

2. Supplemental Results

Cox model

Additional Cox model results are presented in Table S1 and Figure S1.

Time to treatment failure

In exploratory analyses, the time to treatment failure was 38.0 (28.0–68.0) and 28.0 (22.0–38.0) hours in the Intervention group 1 and 2 (respectively), and 2.0 (1.0–23.0) hours in the control group, respectively [Intervention group 1 vs. 2, mean difference (MD) = 14.4hours, 95% confidence interval (CI): −9.8 to 38.6 hours; P = 0.96]; Intervention group 2 vs. control, MD = 16.4hours, 95% CI: 4.9 to 27.9 hours; P = 0.03;Intervention group 1 vs. control, MD=30.8hours, 95% CI: 6.7 to 54.9 hours; P = 0.06; the latter 2 P-values were subjected to Bonferroni correction).

Time points without escalation of support above its initial level

There were more follow-up time points without escalation of support above its initial level and with SpO_2>92% or respiratory rate within 12–20 breaths/min in Intervention group 1 vs. both Intervention group 2 and control (Table S2).

Episodes of hypoxemia in patients without treatment failure

In patients without treatment failure and ≥1 episode of sustained hypoxemia as defined in Methods [Intervention group 1: 13/33 (39.4%), Intervention group 2:18/33 (54.5%), and control: 5/33 (15.2%)], SpO_2 was always promptly restored to >92% by 1) increasing the gas flow of the high-flow nasal canula by 5 to 20 L/min (Intervention group 2) and/or by increasing the administered FiO_2 by 0.1 to 0.3 (all groups); and 2) prescribing additional postoperative analgesia (e.g. 50 to 75 mg of intravenous tramadol) to facilitate deep breathing/voluntary coughing.

Results on non-outcome physiological variables

Results of linear mixed model analysis on the rest of the determined, non-outcome variables are presented in Tables S3 and S4. There was no significant effect of group or group*time on $PaCO_2$, arterial blood lactate, and hemoglobin concentration; (Table S3). There was a significant effect of group on systolic and mean arterial pressure (marginal mean estimates were significantly higher in Intervention group 2 vs. the other 2 groups). No other significant effect of group or group*time was noted on the hemodynamic variables, peripheral body temperature, and the norepinephrine infusion rate (Table S4). Lastly, between-group comparisons of fluid balance at 24 and 48 hours after randomization did not reveal any significant difference (Table S4).

Boxplot graphs

Boxplots showing the time-course of peripheral oxygen saturation and respiratory rate are presented in Figures S2 and S3.

Boxplot presentations of the PaO_2/inspired O_2 fraction (FiO_2), PaO_2, and FiO_2 are provided in Figures S4, S5, and S6.

A boxplot presentation of visual analogue scale comfort scores is provided in Figure S7.

References

1. Mauri T, Alban L, Turrini C, Cambiaghi B, Carlesso E, Taccone P, Bottino N, Lissoni A, Spadaro S, Volta CA, Gattinoni L, Pesenti A, Grasselli G. Optimum support by high-flow nasal cannula in acute hypoxemic respiratory failure: effects of increasing flow rates. Intensive Care Med. 2017;43(10):1453-1463. doi: 10.1007/s00134-017-4890-1.

3. Supplementary Tables

Table S1. Multivariable Cox regression analysis aimed at determining the effect of group on treatment failure.

**Covariate**	**HR**	**95% CI for HR**		
		**Lower**	**Upper**	**P-value**
Group				<0.001
Intervention group 1 (HFNC 60 L/min*) vs. Control	0.11	0.03	0.34	<0.001
Intervention group 2 (HFNC 40 L/min*) vs. Control	0.30	0.12	0.77	0.012
Body mass index (kg/m^2 BSA)	1.09	1.00	1.19	0.04
Euroscore II	1.12	1.00	1.29	0.06
Cardiopulmonary bypass time (min)	1.01	1.01	1.01	0.03
Duration of postoperative sedation (hours)	1.00	0.96	1.03	0.83
Time on PSV plus SBT duration (min)	1.00	1.00	1.00	0.73

HR, hazard ratio; CI, confidence interval; HFNC, high flow nasal canula; BSA, body surface area; EuroSCORE II, European System for Cardiac Operative Risk Evaluation; SBT, spontaneous breathing trial. Time on PSV coincides with the pre-extubation period of assisted breathing, whereas time on SBT coincides with the pre-extubation period of spontaneous breathing. Statistically significant P-values are highlighted in bold script. Collinearity diagnostics: Condition index: 22.0; Variance inflation index: 1.07-1.36. *, Reflects initial HFNC gas flow level.

Table S2. Results on number (%) of follow-up time points with SpO_2 > 92%, and respiratory rate within 12–20 breaths/min, without escalation of support above its initial level.

**SpO_2 >92%**	**No. (%) of follow-up time points within each group**	**P-value**
**Group**		
Intervention 1 (HFNC 60 L/min*) vs. control	296/405 (73.1%) vs. 202/428 (47.2%)	<0.001 †
Intervention 2 (HFNC 40 L/min*) vs. control	170/404 (42.1%) vs. 202/428 (47.2%)	0.14 ‡
Intervention 1 vs. Intervention 2	296/405 (73.1%) vs. 170/404 (42.1%)	<0.001 †
Respiratory rate within 12-20 breaths/min	No. (%) of follow-up time points within each group	P-value
**Group**		
Intervention 1 vs. control	228/405 (56.3%) vs. 169/428 (39.5%)	<0.001 †
Intervention 2 vs. control	145/404 (35.9%) vs. 169/428 (39.5%)	0.32 ‡
Intervention 1 vs. Intervention 2	228/405 (56.3%) vs. 145/404 (39.9%)	<0.001 †

SpO_2, peripheral oxygen saturation; HFNC, high-flow nasal canula. *, Reflects initial HFNC gas flow level. †, Value corrected for multiple comparisons. ‡, Value not corrected for multiple comparisons.

Table S3. Results of mixed-model analysis for $PaCO_2$, arterial blood lactate, and hemoglobin concentration. .

**Dependent variable - $PaCO_2$**	**F**	**P-value**	**AIC**	**% Var.**
Effect of Group (fixed factor)	1.5	0.22	−4451.9	72.7%
Effect of Time (fixed factor)	5.9	<0.001 †		
Effect of Group* Time (interaction)	0.9	0.64		
Group – pairwise comparisons - $PaCO_2$		Estimated Marginal Mean	95% CI Lower	Upper
Intervention 1 (HFNC 60 L/min*) - mmHg		40.0	38.6	41.5
Intervention 2 (HFNC 40 L/min*) - mmHg		41.3	39.8	42.8
Control - mmHg		41.8	40.3	43.4
Dependent variable - Lactate	F	P-value	AIC	% Var.
Effect of Group (fixed factor)	2.4	0.09	−1691.6	64.9%
Effect of Time (fixed factor)	25.9	<0.001 ‡		
Effect of Group* Time (interaction)	0.8	0.80		

Group – pairwise comparisons - Lactate		Estimated Marginal Mean	95% CI Lower	Upper
Intervention 1 (HFNC 60 L/min*) – mmol/L		1.3	1.2	1.4
Intervention 2 (HFNC 40 L/min*) - mmol/L		1.2	1.0	1.3
Control - mmol/L		1.3	1.2	1.4
Dependent variable - Hemoglobin	F	P-value	AIC	% Var.
Effect of Group (fixed factor)	1.7	0.18	−4880.7	84.9%
Effect of Time (fixed factor)	14.4	<0.001§		
Effect of Group* Time (interaction)	1.4	0.10		
Group – pairwise comparisons - Hemoglobin		Estimated Marginal Mean	95% CI Lower	Upper
Intervention 1 (HFNC 60 L/min*) – g/dL		10.4	10.0	10.9
Intervention 2 (HFNC 40 L/min*) – g/dL		9.8	9.4	10.3
Control - mmHg		10.0	9.5	10.4

F, shows the value of the F statistic for the effects of the fixed factors and of their interaction; $PaCO_2$, carbon dioxide arterial partial pressure; FiO_2, inspired oxygen fraction; AIC, Akaike's information criterion for goodness of fit; % Var., percent variance (of the observed values) explained by the linear mixed model estimates; CI, confidence interval; HFNC, high-flow nasal canula. Logarithmic transformation of lactate and hemoglobin data was reversed for the purpose of numeric presentation; logarithmic transformation of $PaCO_2$ data was not required as their distribution did not deviate significantly from normality by the Kolmogorov-Smirnov test (see also Statistical Analysis) *, Reflects initial HFNC gas flow level. †, Bonferroni-corrected pairwise comparisons revealed that the mean estimates for PaCO2 of the total study population exhibited significant drops at ≥32 hours relative to ≤20 hours postextubation. ‡, Bonferroni-corrected pairwise comparisons revealed that the mean estimates for arterial blood lactate of the total study population exhibited significant drops at ≥8 hours relative to ≤2 hours postextubation. §, Bonferroni-corrected pairwise comparisons revealed that the mean estimates for hemoglobin concentration of the total study population exhibited significant drops at ≥16 hours relative to ≤4 hours post-extubation.

Table S4. Results of mixed-model analysis for hemodynamic variables, norepinephrine infusion rate and frequency of norepinephrine use at the time points of follow-up, and fluid balance at 24 and 48 hours after randomization.

**Dependent variable – SAP**	**F**	**P-value**	**AIC**	**% Var.**
Effect of Group (fixed factor)	7.8	0.001	−3896.6	42.5%
Effect of Time (fixed factor)	1.2	0.28		
Effect of Group* Time (interaction)	0.6	0.93		
Group – pairwise comparisons - SAP		Estimated Marginal Mean	95% CI Lower	Upper
Intervention 1 (HFNC 60 L/min*) - mmHg		118.8	115.9	121.7
Intervention 2 (HFNC 40 L/min*) - mmHg		125.9 †,‡	122.9	129.0
Control - mmHg		118.3	115.5	121.3
Dependent variable - DAP	F	P-value	AIC	% Var.
Effect of Group (fixed factor)	0.6	0.54	−3463.0	51.6%
Effect of Time (fixed factor)	2.7	0.002 §		
Effect of Group* Time (interaction)	0.8	0.77		
Group – pairwise comparisons - DAP		Estimated Marginal Mean	95% CI Lower	Upper
Intervention 1 (HFNC 60 L/min*) – mmHg		59.0	56.9	61.2
Intervention 2 (HFNC 40 L/min*) - mmHg		59.5	57.3	61.7
Control - mmHg		57.9	55.8	60.0
Dependent variable - MAP	F	P-value	AIC	% Var.
Effect of Group (fixed factor)	3.8	0.03	−4015.6	41.2%
Effect of Time (fixed factor)	2.0	0.03**		
Effect of Group* Time (interaction)	0.8	0.79		
Group – pairwise comparisons - MAP		Estimated Marginal Mean	95% CI Lower	Upper
Intervention 1 (HFNC 60 L/min*) – mmHg		79.1	77.2	81.0
Intervention 2 (HFNC 40 L/min*) – mmHg		81.8 ††	79.9	83.8
Control - mmHg		78.2	76.4	80.1

Table S4. (continued).

**Dependent variable – Heart Rate**	**F**	**P-value**	**AIC**	**% Var.**
Effect of Group (fixed factor)	0.9	0.40	−3595.1	50.2%
Effect of Time (fixed factor)	4.3	<0.001 ‡‡		
Effect of Group* Time (interaction)	0.7	0.88		
Group – pairwise comparisons - Heart Rate	Estimated Marginal Mean		95% CI Lower	Upper
Intervention 1 (HFNC 60 L/min*) – beats/min	86.0		83.2	89.0
Intervention 2 (HFNC 40 L/min*) – beats/min	83.3		80.5	86.2
Control - beats/min	84.0		81.2	86.9
Dependent variable – CVP	F	P-value	AIC	% Var.
Effect of Group (fixed factor)	0.1	0.93	−281.9	13.8%
Effect of Time (fixed factor)	2.0	0.03§§		
Effect of Group* Time (interaction)	0.9	0.64		
Group – pairwise comparisons - CVP	Estimated Marginal Mean		95% CI Lower	Upper
Intervention 1 (HFNC 60 L/min *) – mmHg	9.1		7.6	10.9
Intervention 2 (HFNC 40 L/min *) – mmHg	9.0		7.4	11.1
Control - µg/kg/min	8.7		7.1	10.7
Dependent variable – Temperature	F	P-value	AIC	% Var.
Effect of Group (fixed factor)	0.8	0.48	−9167.6	51.2%
Effect of Time (fixed factor)	1.8	0.048 ***		
Effect of Group* Time (interaction)	0.9	0.62		
Group – pairwise comparisons - Temperature	Estimated Marginal Mean		95% CI Lower	Upper
Intervention 1 (HFNC 60 L/min *) – Degrees Celcius	36.8		36.7	37.0
Intervention 2 (HFNC 40 L/min *) – Degrees Celcius	37.0		36.8	37.1
Control - µg/kg/min	36.9		36.8	37.0

Table S4. (continued).

**Dependent variable – NE infusion rate †††**	**F**	**P-value**	**AIC**	**% Var.**
Effect of Group (fixed factor)	0.3	0.73	−87.7	82.0%
Effect of Time (fixed factor)	9.0	0.03 ‡‡‡		
Effect of Group* Time (interaction)	2.1	0.36		
Group – pairwise comparisons - NE infusion rate †††	Estimated Marginal Mean		95% CI Lower	Upper
Intervention 1 (HFNC 60 L/min *) – µg/kg/min	0.06		0.04	0.09
Intervention 2 (HFNC 40 L/min *) – µg/kg/min	0.05		0.02	0.08
Control - µg/kg/min	0.05		0.03	0.07
Fluid balance (mL) at 24 hours after randomization	Mean±SD			P-value
Intervention 1 (HFNC 60 L/min *) vs. Control	535±274 vs. 529±239			0.92
Intervention 2 (HFNC 40 L/min *) vs. Control	643±280 vs. 529±239			0.08
Intervention 1 vs. Intervention 2	535±274 vs. 643±280			0.12
Fluid balance (mL) at 48 hours after randomization	Mean±SD			P-value
Intervention 1 (HFNC 60 L/min *) vs. Control	1068±526 vs. 1075±509			0.96
Intervention 2 (HFNC 40 L/min *) vs. Control	1289±527 vs. 1075±509			0.10
Intervention 1 vs. Intervention 2	1068±526 vs. 1289±527			0.09

F, shows the value of the F statistic for the effects of the fixed factors and of their interaction; SAP, systolic intra-arterial pressure; AIC, Akaike's information criterion for goodness of fit; % Var., percent variance (of the observed values) explained by the linear mixed model estimates; CI, confidence interval; HFNC, high-flow nasal canula; DAP, diastolic intra-arterial pressure; MAP, mean arterial pressure; HR, heart rate; CVP, central venous pressure; NE, norepinephrine. Logarithmic transformation of SAP, DAP, MAP, CVP, (peripheral body) Temperature, and NE data was reversed for the purpose of numeric presentation (see also Statistical Analysis) *, Reflects initial HFNC gas flow level. †, P = 0.004 vs. Intervention 1 ‡, P= 0.002 vs. Control; §, Bonferroni-corrected pairwise comparisons of overall mean estimates for DAP at postextubation follow-up time points did not reveal any significant difference. **, Bonferroni-corrected pairwise comparisons of overall mean estimates for MAP at postextubation follow-up time points did not reveal any significant difference. ††, P= 0.03 vs. Control;

‡‡, Bonferroni-corrected pairwise comparisons revealed that the mean estimate for heart rate of the total study population exhibited significant drops at ≥20 hours relative to ≤1 hour after extubation. §§, Bonferroni-corrected pairwise comparisons of overall mean estimates for CVP at postextubation follow-up time points did not reveal any significant difference. ***, Bonferroni-corrected pairwise comparisons of overall mean estimates for peripheral body temperature at postextubation follow-up time points did not reveal any significant difference. †††, Data originate from 18, 10, and 17 Intervention group 1, Intervention group 2, and control patients, respectively; these patients required norepinephrine at at least 1 follow-up time-point, in order to maintain a mean arterial pressure of ≥70 mmHg. ‡‡‡, Bonferroni-corrected pairwise comparisons of mean estimates for norepinephrine infusion rate of all patients who required vasopressor support were significantly higher within 10 to 28 hours postextubation relative to the first follow-up time point (just after extubation).

4. Supplementary Figures

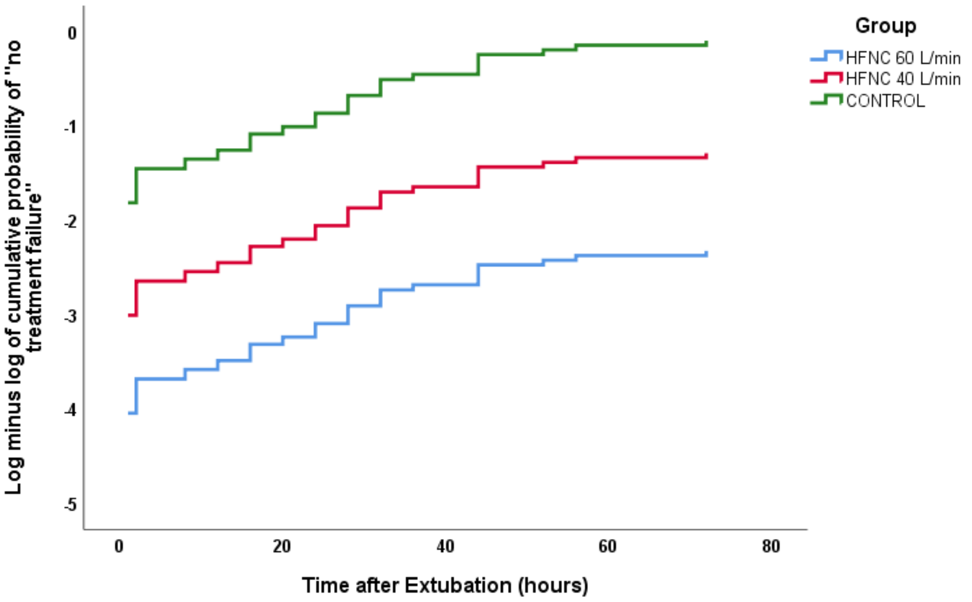

No. at risk HFNC 60 L/min*	33	33	31	30	29
HFNC 40 L/min*	33	31	25	23	23
CONTROL	33	20	18	15	15

Figure S1. Log minus log plot of the cumulative probability of the absence of treatment failure, i.e. ″no treatment failure″ in Intervention group 1 (HFNC 60 L/min), Intervention group 2 (HFNC 40 L/min), and Control group. HFNC, high-flow nasal canula. Curves are parallel on visual inspection, thereby supporting the proportional hazards assumption, i.e. that ″hazard for any individual = fixed proportion of hazard for any other individual.″ * Reflects initial HFNC gas flow level.

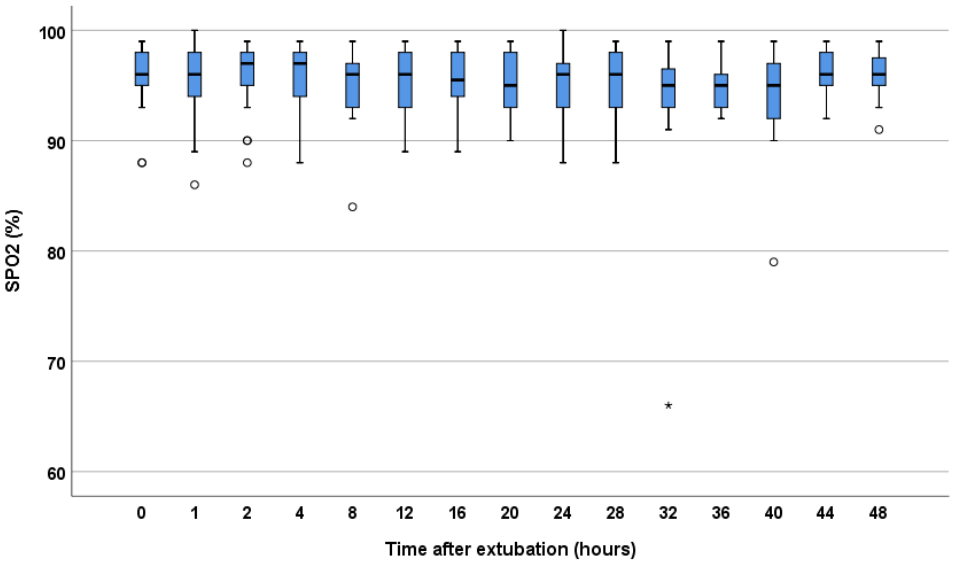

33	33	33	33	33	33	32	29	23	26	23	23	18	18	15

(**a**)

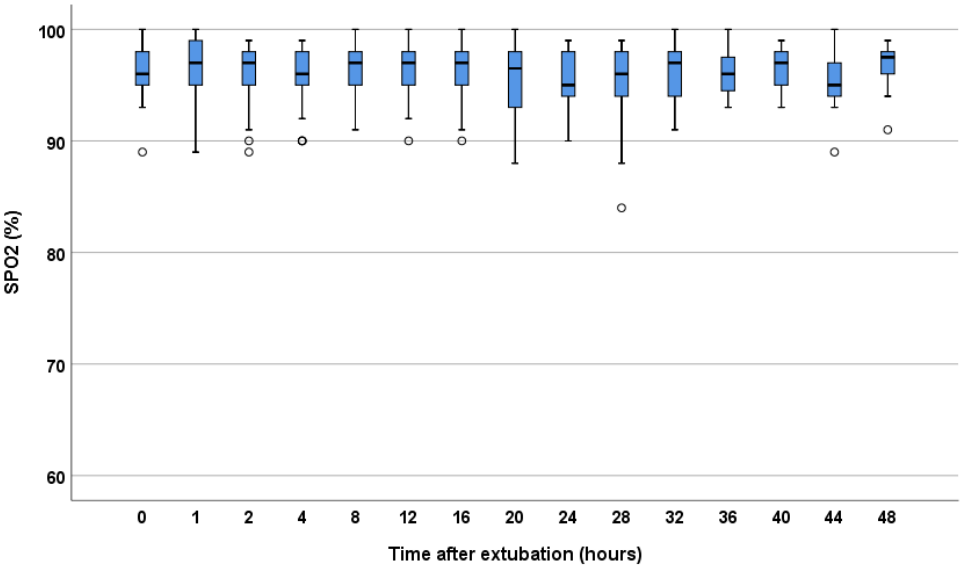

33	33	33	33	33	33	32	30	29	25	21	24	14	17	14

(**b**)

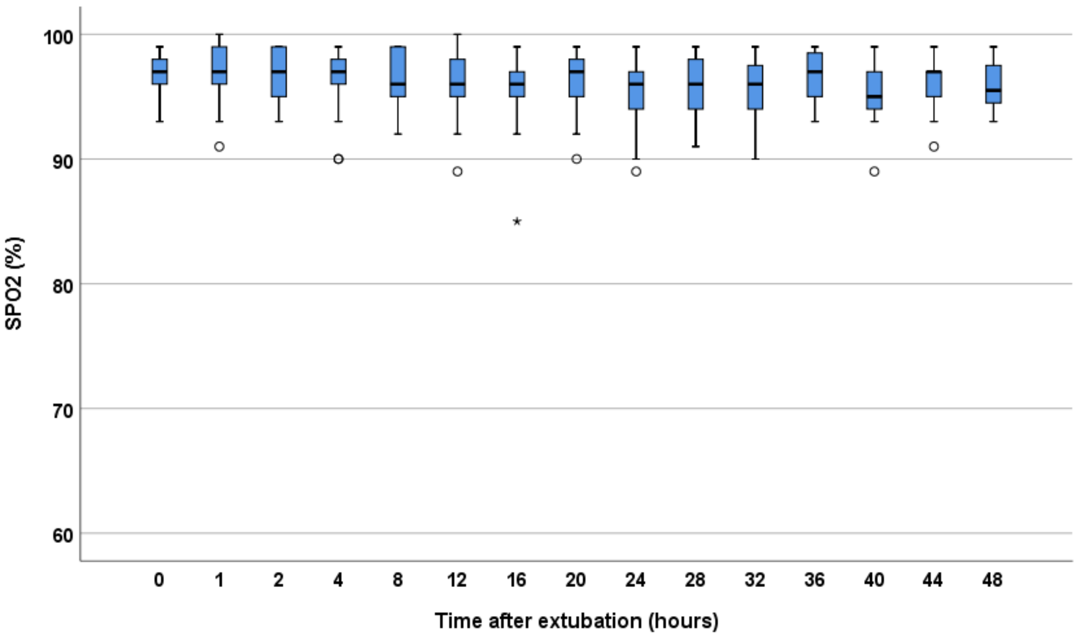

33	33	33	33	33	33	33	29	27	27	27	23	23	21	20

(c)

Figure S2. Boxplots of peripheral oxygen saturation (SPO2) (%) (over the first 48 hours after extubation. Numbers below each one of the plots represent numbers of patients participating in the analyses at the corresponding time points. **(a):** Intervention group 1, high-flow-nasal canula (HFNC) support starting at 60 L/min and an FiO_2 of 0.6. **(b):** Intervention group 2, HFNC support starting at 40 L/min and an FiO_2 of 0.6. **(c):** Control group, conventional oxygen therapy via a Venturi mask delivering an FiO_2 of 0.6. Horizontal lines represent median value; box height reflects intraquartile range; bars on the top and bottom of the boxes and/or symbols (i.e. circles and stars) reflect range of values.

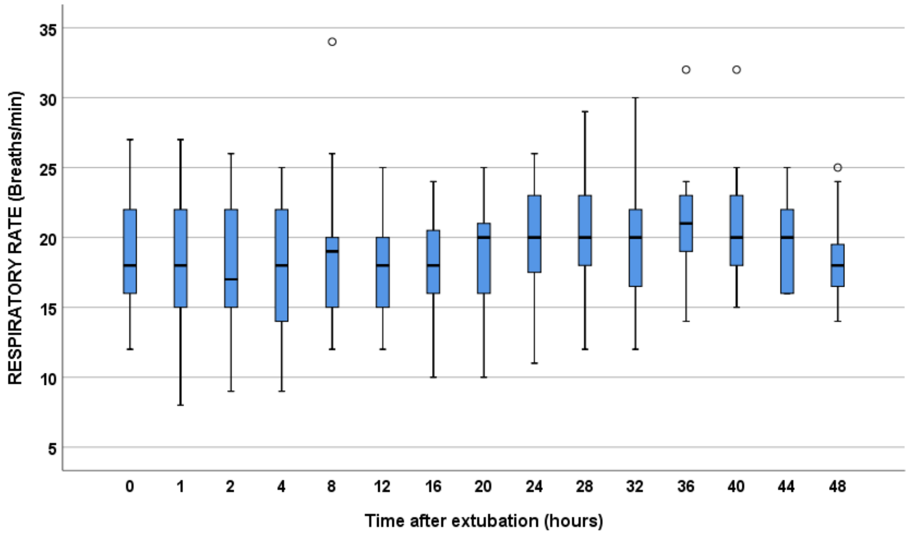

33	33	33	33	33	33	32	29	23	26	23	23	18	18	15

(a)

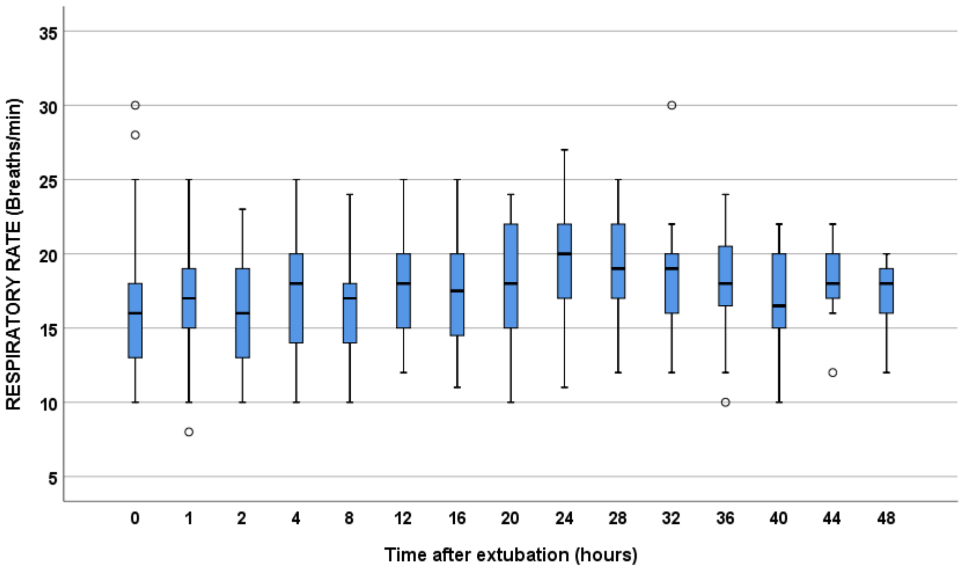

33	33	33	33	33	33	32	30	29	25	21	24	14	17	14

(b)

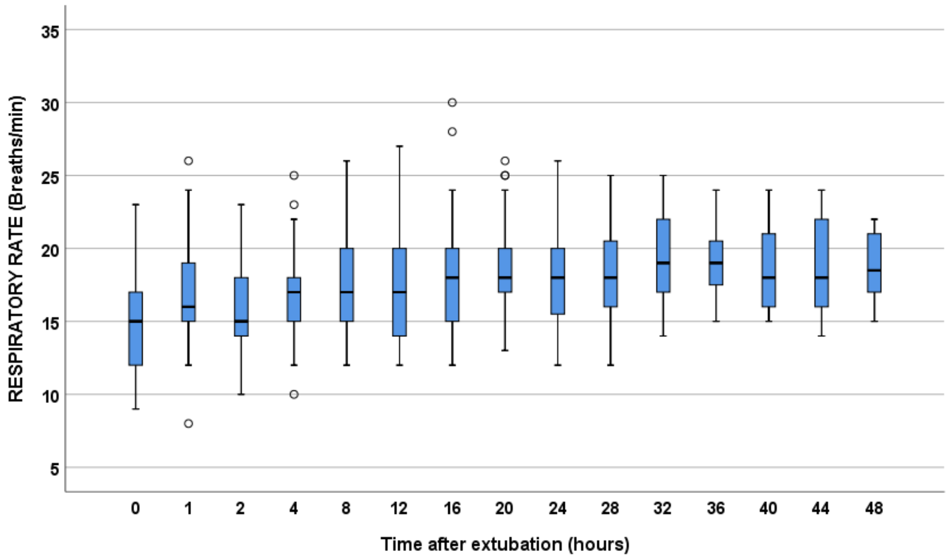

33	33	33	33	33	33	33	29	27	27	27	23	23	21	20

(c)

Figure S3. Boxplots of respiratory rate (breaths/min) over the first 48 hours after extubation. Numbers below each one of the plots represent numbers of patients participating in the analyses at the corresponding time points. **(a):** Intervention group 1, high-flow-nasal canula (HFNC) support starting at 60 L/min and a fraction of inspired oxygen (FiO_2) of 0.6. **(b):** Intervention group 2, HFNC support starting at 40 L/min and an FiO_2 of 0.6. **(c):** Control group, conventional oxygen therapy via a Venturi mask delivering an FiO_2 of 0.6. Horizontal lines represent median value; box height reflects intraquartile range; bars on the top and bottom of the boxes and/or symbols (i.e. circles and stars) reflect range of values.

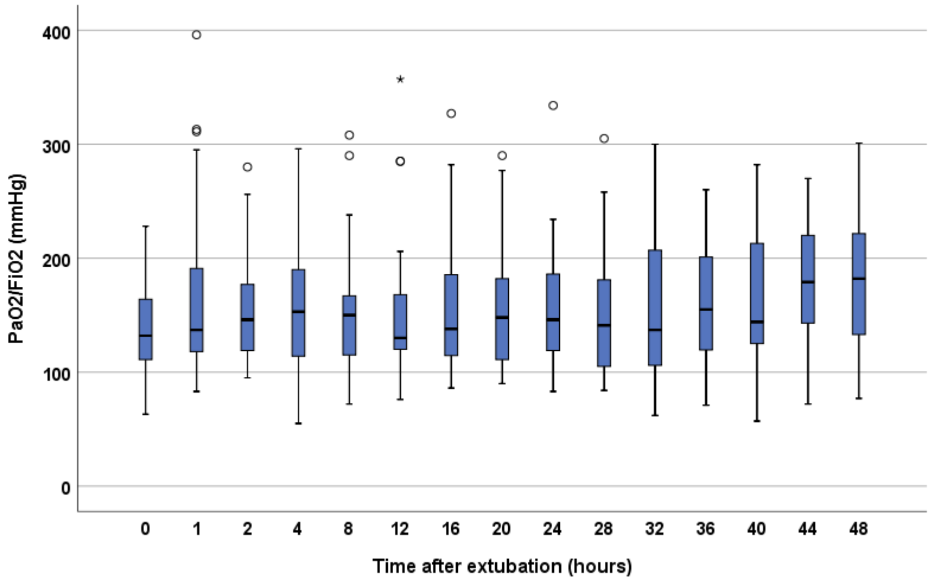

33	33	33	33	33	33	32	29	22	26	22	23	17	18	15

(a)

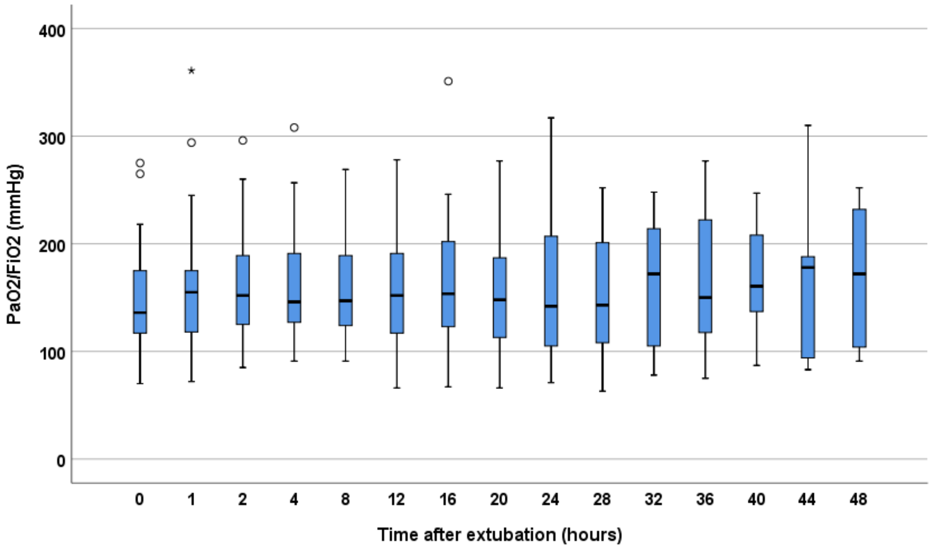

33	33	33	33	33	33	32	30	29	25	21	24	14	17	14

(b)

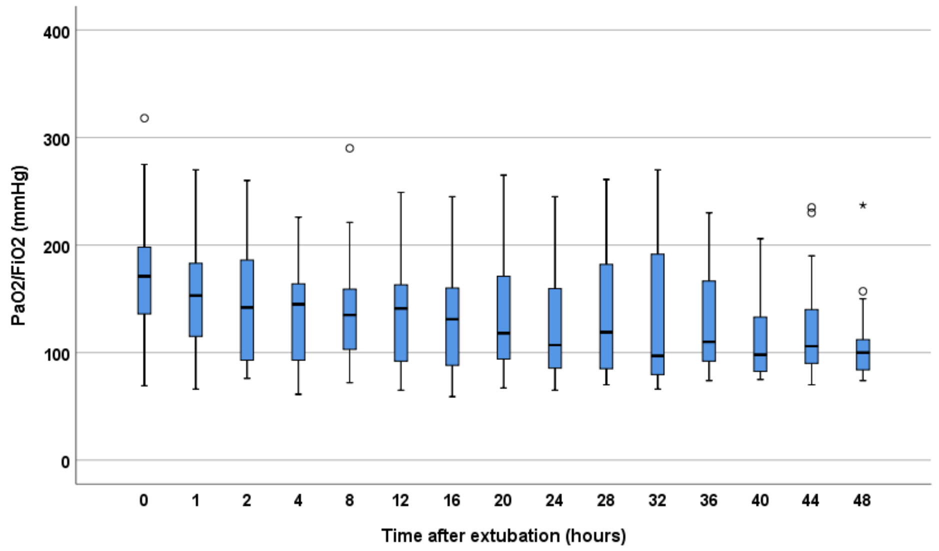

33	33	33	33	33	33	33	29	27	27	27	23	23	21	20

(c)

Figure S4. Boxplots of PaO2/fraction of inspired oxygen (FiO2) over the first 48 hours after extubation. Numbers below each one of the plots represent numbers of patients participating in the analyses at the corresponding time points. **(a):** Intervention group 1, high-flow-nasal canula (HFNC) support starting at 60 L/min and an FiO2 of 0.6. **(b):** Intervention group 2, HFNC support starting at 40 L/min and an FiO2 of 0.6. **(c):** Control group, conventional oxygen therapy via a Venturi mask delivering an FiO2 of 0.6. Horizontal lines represent median value; box height reflects intraquartile range; bars on the top and bottom of the boxes and/or symbols (i.e. circles and stars) reflect range of values.

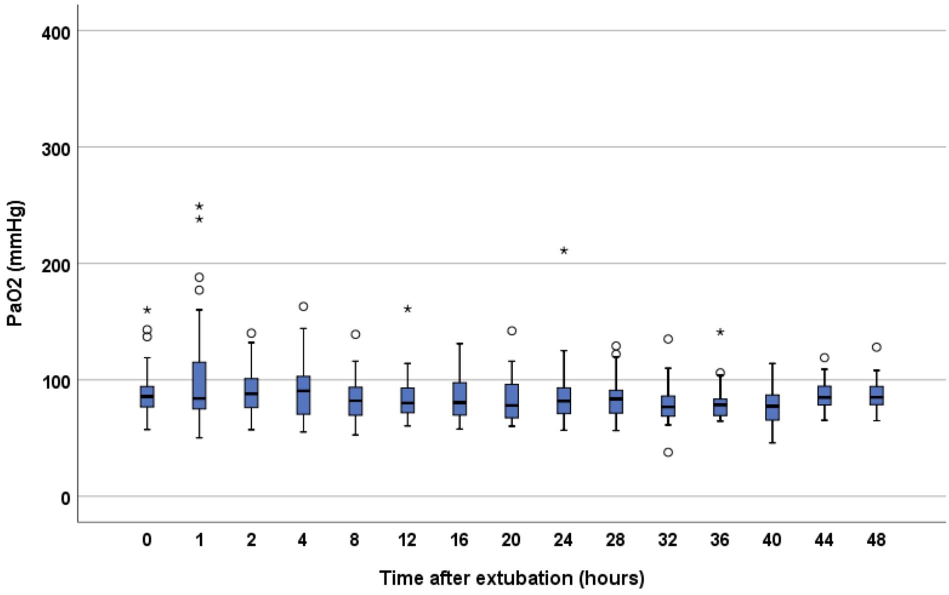

33	33	33	33	33	33	32	29	22	26	22	23	17	18	15

(a)

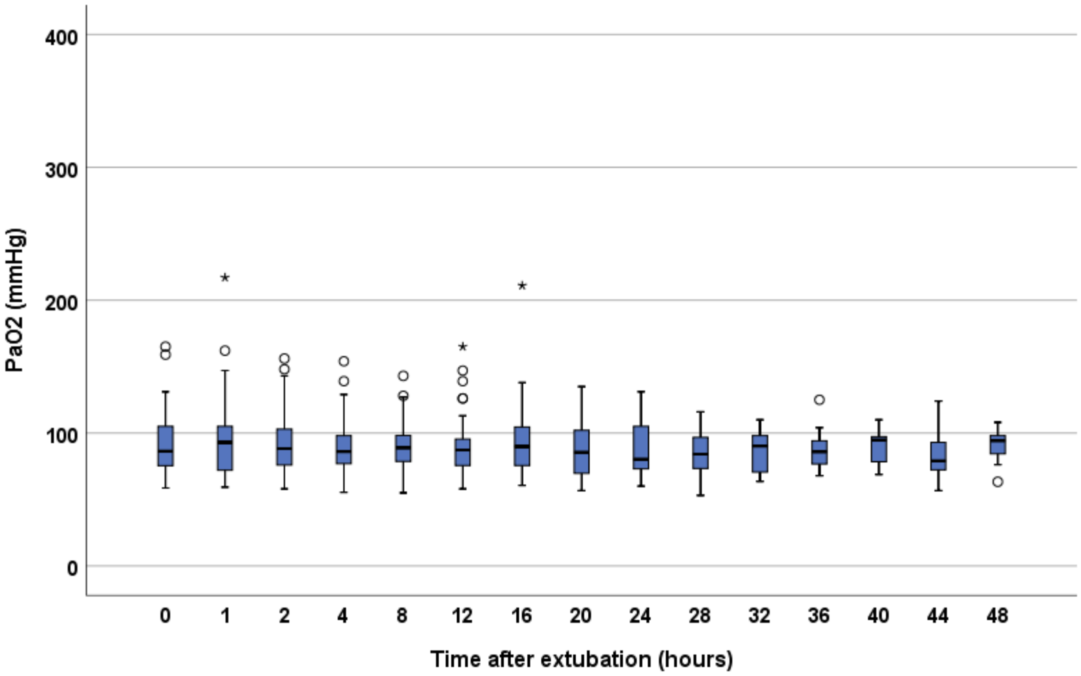

33	33	33	33	33	33	32	30	29	25	21	24	14	17	14

(b)

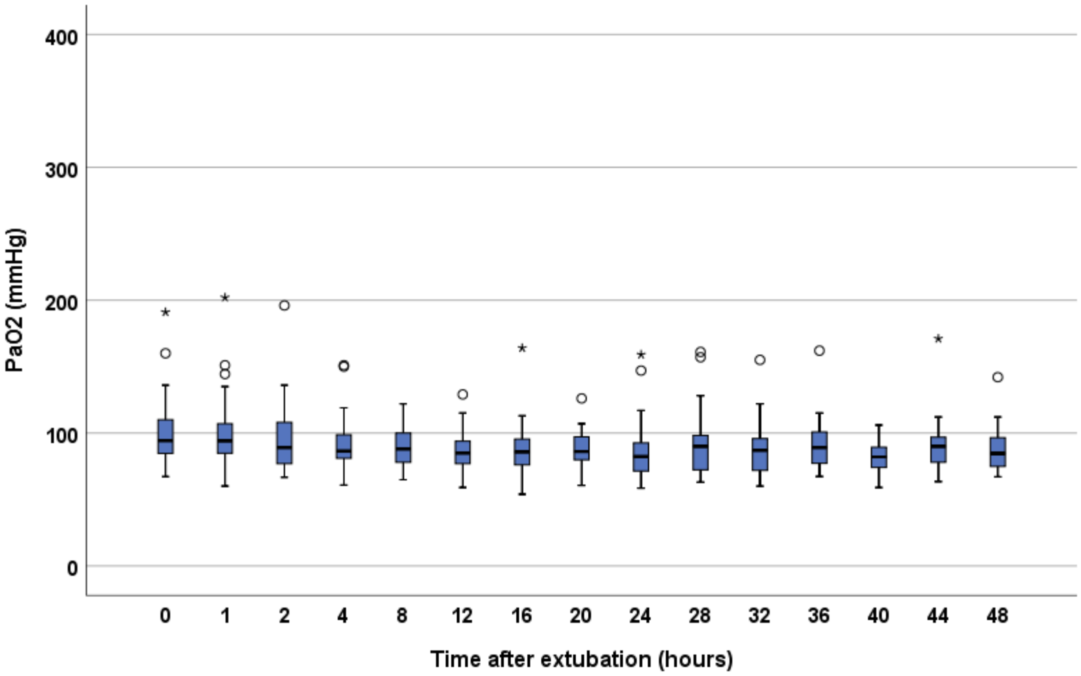

33	33	33	33	33	33	33	29	27	27	27	23	23	21	20

(c)

Figure S5. Boxplots of PaO_2 over the first 48 hours after extubation. Numbers below each one of the plots represent numbers of patients participating in the analyses at the corresponding time points. **(a):** Intervention group 1, high-flow-nasal canula (HFNC) support starting at 60 L/min and an FiO_2 of 0.6. **(b):** Intervention group 2, HFNC support starting at 40 L/min and an FiO_2 of 0.6. **(c):** Control group, conventional oxygen therapy via a Venturi mask delivering an FiO_2 of 0.6. Horizontal lines represent median value; box height reflects intraquartile range; bars on the top and bottom of the boxes and/or symbols (i.e. circles and stars) reflect range of values.

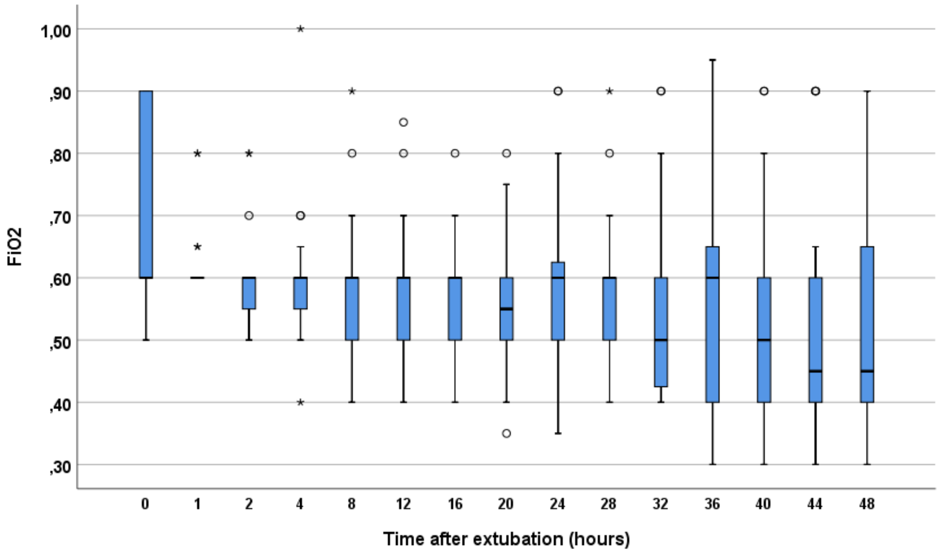

33	33	33	33	33	33	32	29	23	26	23	23	18	18	15

(a)

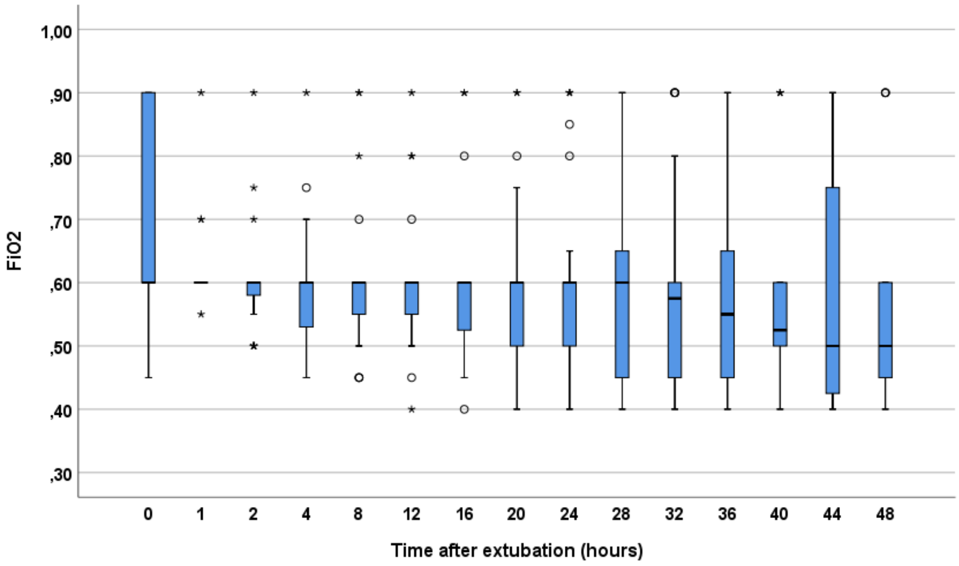

33	33	33	33	33	33	32	30	29	25	22	24	14	17	14

(b)

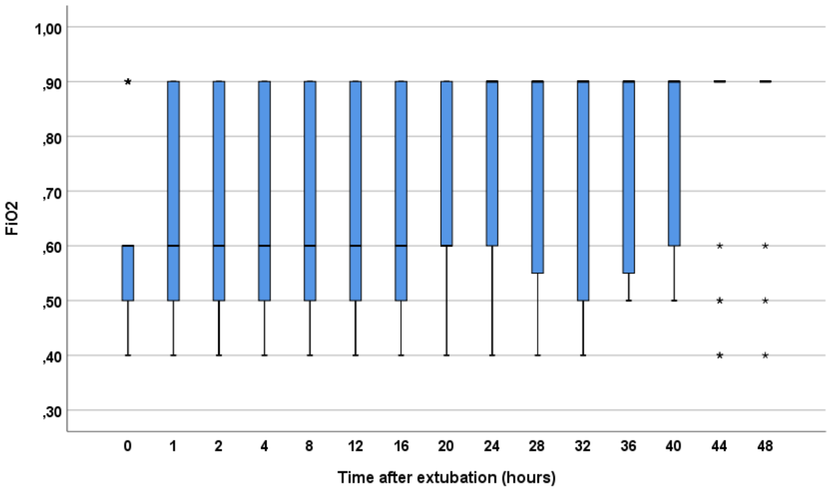

33	33	33	33	33	33	33	29	27	27	27	23	23	21	20

(c)

Figure S6. Boxplots of the fraction of inspired oxygen (FiO_2) over the first 48 hours after extubation. Numbers below each one of the plots represent numbers of patients participating in the analyses at the corresponding time points. **(a):** Intervention group 1, high-flow-nasal canula (HFNC) support starting at 60 L/min and an FiO_2 of 0.6. **(b):** Intervention group 2, HFNC support starting at 40 L/min and an FiO_2 of 0.6. **(c):** Control group, conventional oxygen therapy via a Venturi mask delivering an FiO_2 of 0.6. Horizontal lines represent median value; box height reflects intraquartile range; bars on the top and bottom of the boxes and/or symbols (i.e. circles and stars) reflect range of values.

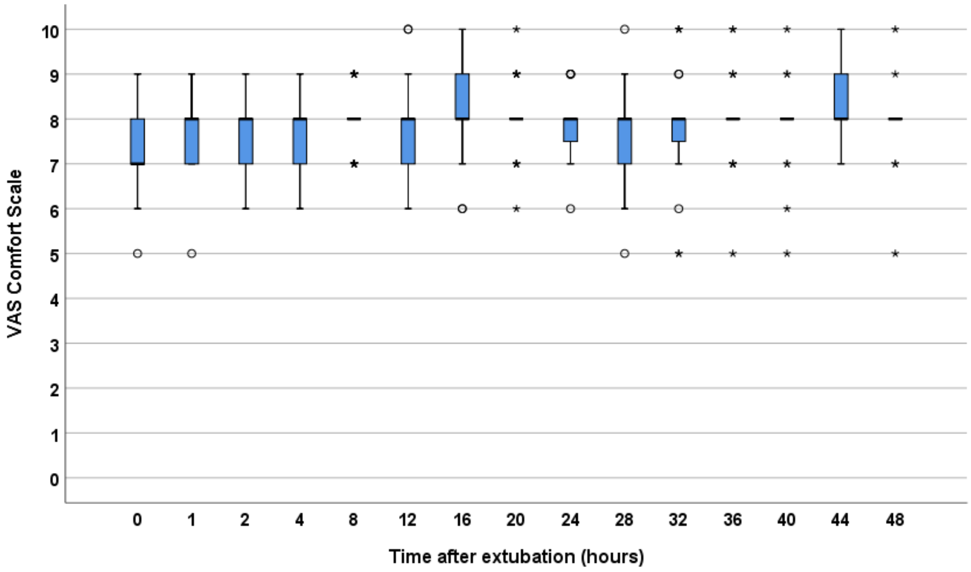

32	33	33	33	33	33	32	29	23	26	23	23	18	18	15

(a)

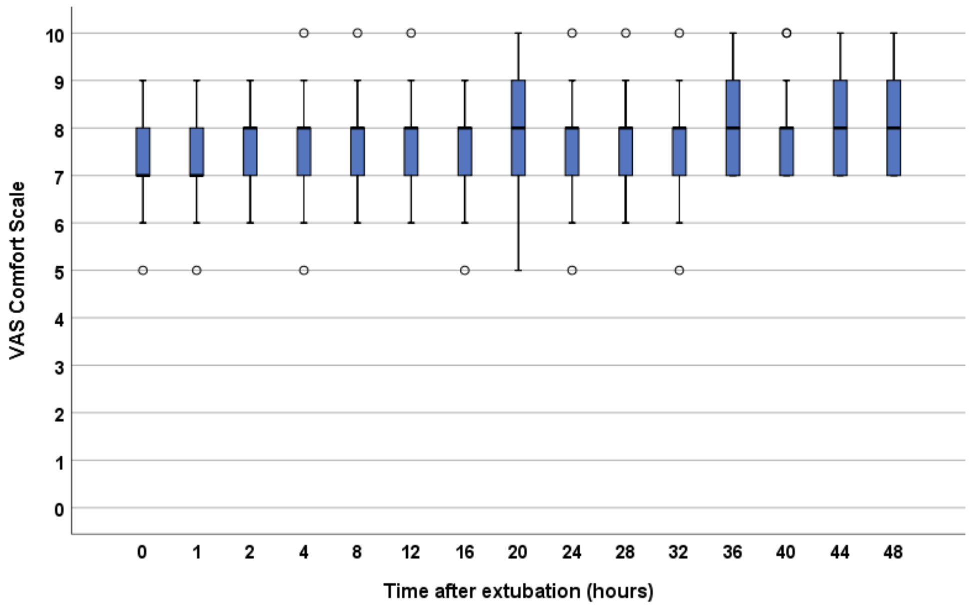

33	33	33	33	33	33	32	30	29	25	21	24	14	17	14

(b)

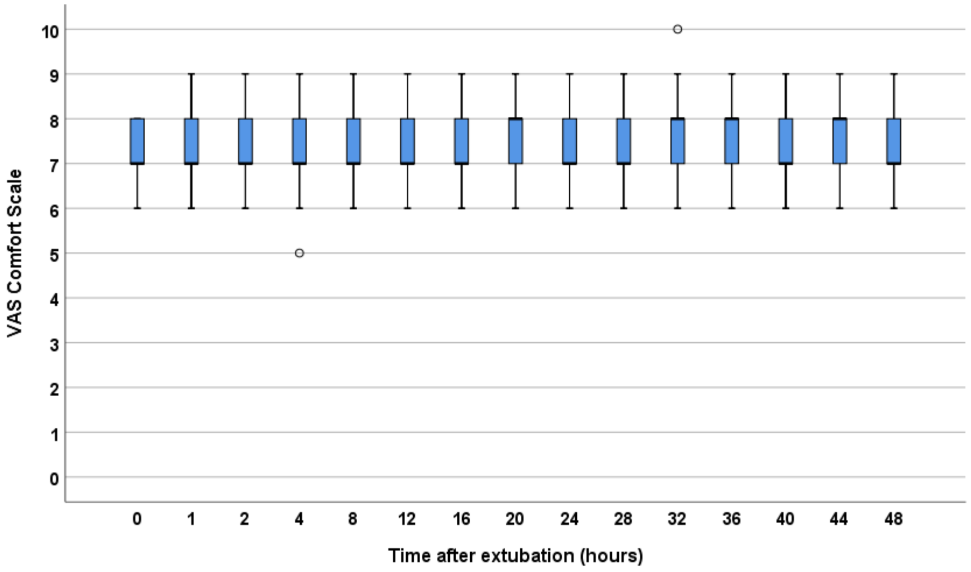

33	33	33	33	33	33	30	29	27	27	27	23	23	22	20

(c)

Figure S7. Boxplots of the Visual Analogue Scale (VAS) Comfort Score over the first 48 hours after extubation. Numbers below each one of the plots represent numbers of patients participating in the analyses at the corresponding time points. **(a):** Intervention group 1, high-flow-nasal canula (HFNC) support starting at 60 L/min and an FiO_2 of 0.6. **(b):** Intervention group 2, HFNC support starting at 40 L/min and an FiO_2 of 0.6. **(c):** Control group, conventional oxygen therapy via a Venturi mask delivering an FiO_2 of 0.6. Horizontal lines represent median value; box height reflects intraquartile range; bars on the top and bottom of the boxes and/or symbols (i.e. circles and stars) reflect range of values.

CONSORT 2010 checklist of information to include when reporting a randomised trial*.

Section/Topic	Item No	Checklist item	Reported on page No
		**Title and abstract**	
	1a	Identification as a randomised trial in the title	1
	1b	Structured summary of trial design, methods, results, and conclusions (for specific guidance see CONSORT for abstracts)	ABSTRACT written according to Guide for Authors
		**Introduction**	
Background and objectives	2a	Scientific background and explanation of rationale	3
	2b	Specific objectives or hypotheses	3
		**Methods**	
Trial design	3a	Description of trial design (such as parallel, factorial) including allocation ratio	4, 5
	3b	Important changes to methods after trial commencement (such as eligibility criteria), with reasons	NOT APPLICABLE
Participants	4a	Eligibility criteria for participants	4, 5
	4b	Settings and locations where the data were collected	4
Interventions	5	The interventions for each group with sufficient details to allow replication, including how and when they were actually administered	5, 6
Outcomes	6a	Completely defined pre-specified primary and secondary outcome measures, including how and when they were assessed	6, 7
	6b	Any changes to trial outcomes after the trial commenced, with reasons	NOT APPLICABLE
Sample size	7a	How sample size was determined	7
	7b	When applicable, explanation of any interim analyses and stopping guidelines	7
Randomisation:			
Sequence generation	8a	Method used to generate the random allocation sequence	5
	8b	Type of randomisation; details of any restriction (such as blocking and block size)	5

Allocation concealment mechanism	9	Mechanism used to implement the random allocation sequence (such as sequentially numbered containers), describing any steps taken to conceal the sequence until interventions were assigned	5
Implementation	10	Who generated the random allocation sequence, who enrolled participants, and who assigned participants to interventions	5
Blinding	11a	If done, who was blinded after assignment to interventions (for example, participants, care providers, those assessing outcomes) and how	THE STUDY WAS UNBLINDED, 4
	11b	If relevant, description of the similarity of interventions	5, 6, 22
Statistical methods	12a	Statistical methods used to compare groups for primary and secondary outcomes	7, 8
	12b	Methods for additional analyses, such as subgroup analyses and adjusted analyses	7, 8
		**Results**	
Participant flow (a diagram is strongly recommended)	13a	For each group, the numbers of participants who were randomly assigned, received intended treatment, and were analysed for the primary outcome	9, FIGURE 1
	13b	For each group, losses and exclusions after randomisation, together with reasons	NOT APPLICABLE
Recruitment	14a	Dates defining the periods of recruitment and follow-up	9
	14b	Why the trial ended or was stopped	NOT APPLICABLE
Baseline data	15	A table showing baseline demographic and clinical characteristics for each group	10,11, TABLE 1
Numbers analysed	16	For each group, number of participants (denominator) included in each analysis and whether the analysis was by original assigned groups	8, 9, FIGURE 1
Outcomes and estimation	17a	For each primary and secondary outcome, results for each group, and the estimated effect size and its precision (such as 95% confidence interval)	11-19, FIGURE 2, TABLES 2-4; TABLE S1, TABLES 2-4
	17b	For binary outcomes, presentation of both absolute and relative effect sizes is recommended	13-15; TABLE 2; TABLE S1 & S2
Ancillary analyses	18	Results of any other analyses performed, including subgroup analyses and adjusted analyses, distinguishing pre-specified from exploratory	19-21; TABLE 5SUPPLEMENT, pages 2-4, TABLES S2-S4, FIGURES S2-S7
Harms	19	All important harms or unintended effects in each group (for specific guidance see CONSORT for harms)	19-21; TABLE 5

		**Discussion**	
Limitations	20	Trial limitations, addressing sources of potential bias, imprecision, and, if relevant, multiplicity of analyses	25
Generalisability	21	Generalisability (external validity, applicability) of the trial findings	25
Interpretation	22	Interpretation consistent with results, balancing benefits and harms, and considering other relevant evidence	22-25
		**Other information**	
Registration	23	Registration number and name of trial registry	4
Protocol	24	Where the full trial protocol can be accessed, if available	Submitted to Clinicaltrials.gov
Funding	25	Sources of funding and other support (such as supply of drugs), role of funders	26, 27
